# Disposal habits and microbial load of solid medical waste in sub-district healthcare facilities and households in Yilo-Krobo municipality, Ghana

**DOI:** 10.1371/journal.pone.0261211

**Published:** 2021-12-10

**Authors:** Fredrick Egbenyah, Emilia Asuquo Udofia, Jesse Ayivor, Mary-Magdalene Osei, John Tetteh, Patience B. Tetteh-Quarcoo, Eric Sampane-Donkor

**Affiliations:** 1 Institute of Environment and Sanitation Studies, College of Basic and Applied Sciences, University of Ghana, Legon, Accra, Ghana; 2 Department of Community Health, University of Ghana Medical School, College of Health Sciences, University of Ghana, Korle Bu, Accra, Ghana; 3 Department of Medical Microbiology, University of Ghana Medical School, College of Health Sciences, University of Ghana, Korle Bu, Accra, Ghana; Texas A&M University System, QATAR

## Abstract

The study aimed to assess disposal practices and quantify the microbial load present in SMW from ten sub-district level healthcare facilities and 385 households in Yilo Krobo municipality, Ghana. Disposal of solid medical waste (SMW) was assessed by questionnaire-based surveys, unstructured interviews and field observations. Microbiological analysis identified species and counts of bacteria present in SMW from both sources. Sociodemographic factors influencing the method of SMW disposal in households were evaluated using logistic regression analysis, with statistical significance set at p<0.05. Open burning (29%), burying (25%) and disposal at a dumpsite (49%) were common methods used by households to discard SMW. SMW disposal at a dumpsite was associated with age of respondents in households. Older people (50+ years) were three times more likely to place SMW in household waste later discarded at a dumpsite, compared to younger persons (20–30 years) [a0R, 95%CI = 3.37, 1.41–8.02]. In sub-district level healthcare facilities, open burning and burying were the most common methods used. *Bacillus subtilis*, *Klebsiella pneumonia*, *Pseudomonas aeruginosa*, *Clostridium tetani*, *Enterococcus faecalis*, *Acinetobacter spp*. *Escherichia coli*, *Bacillus cereus and Enterococcus faecium*) were bacteria identified in SMW recovered from both the healthcare facilities and the households. *Klebsiella pneumoniae*, *Acinetobacter spp*. and *Clostridium tetani* were found exclusively in untreated SMW generated in the healthcare facilities. *Bacillus spp*. and *Pseudomonas spp*. were found in one sample of treated SMW. The microbial load in SMW from healthcare facilities and households ranged from 0.036 x 10^3^cfc/mg to 0.167 x 10^3^ cfc/mg and from 0.118 x 10^3^cfc/mg to 0.125 x 10^3^cfc/mg respectively. This highlights the need for institutionalizing appropriate treatment methods in sub-district level facilities or strengthening the linkages with higher level facilities to ensure regular and adequate treatment of SMW. Public guidance on management of SMW generated in households which is context specific should also be provided.

## Introduction

According to the World Health Organization (WHO), medical waste has been defined as an end product of medical services and includes items such as medical devices, sharps, blood, body parts, chemicals, pharmaceuticals, and radioactive materials [1, 2]. Health care facilities are the main source of medical waste, but other sources include household and communal settings where health care activities conducted in these settings also generate medical waste. Globally, it has been estimated that 5.2 million people, including 4 million children, die annually from waste-related diseases and the situation is likely to get worse if appropriate measures are not developed to curb it [1]. There is a growing awareness about solid medical waste (SMW) and its associated hazards, but its management has not received prioritized attention in developing countries [3].

Although hazardous waste constitutes 10% to 25% of the SMW stream, it represents an immediate threat to humans through the transmission of pathogens from biological fluids [4]. Examples of hazardous waste include soiled and/or blood-stained gauze bandages, used lancets and syringes, used and discarded specimen containers, cultures, stocks of infectious agents and human tissue. If SMW is not treated in a way that destroys the pathogenic organisms (viruses, bacteria, parasites or fungi), these pathogens will be present in harmful quantities. They enter the body through punctures and other breaks in the skin, mucous membranes in the mouth, by inhalation, ingestion, or transmission by a vector organism [4]. People who come in direct contact with the waste are at greatest risk. Sharps waste pose a specific hazard through cuts and punctures. The healthcare workers or waste handlers who handle sharps waste can become infected with HIV/AIDS and hepatitis B and C viruses through pricks or reuse of syringes/needles [5]. Commonly identified bacterial pathogens such as *Pseudomonas* spp., *Corynebacterium diphtheriae*, *Escherichia coli*, *Staphylococcus* spp. which are known to cause respiratory tract infections and other diseases have been reported in SMW and should be carefully controlled to prevent associated nosocomial infection [6]. Some of these bacteria exhibit resistance to antibiotics. Drugs that were used to treat associated diseases are now losing their impact due to emerging drug resistant microorganisms including *Escherichia coli* and *Klebsiella pneumonia* [6]. This resistance threatens the effective control against these bacteria that cause UTI, pneumonia and bloodstream infections, resulting in longer hospital stay and higher costs of care. Pathogens present in untreated waste can also leach out and contaminate the soil and surface water [7].

A previous study conducted on samples of biomedical waste from five primary healthcare centres in Edo South, Nigeria, indicated a high prevalence of *Escherichia coli* (39%) and *Staphylococcus aureus* (32%) and a lower prevalence of *Klebsiella pneumonia* (10%) and *Bacillus subtilis* (4%) [8]. Another study conducted in India reported similar species but with a lower prevalence: *Escherichia coli* (15%), *Bacillis subtilis* (12%), *Staphylococcus aureus* (9%) and *Klebsiella pneumonia* (6%). The microbial load ranged from 1.6 x 10^7^ to 3.6 x 10^7^ CFU/gm [9]. In a report by [10], *Klebsiella pneumonia* (71.8%) and *Staphylococcus aureus* (28.2%) were dominant species in SMW with microbial load ranging between 1.15 x 10^14^ to 9.16 x 10^13^ cfu/100g. *Klebsiella species* were dominant in residential areas and microbial loads were higher in residential areas closer to the health facility than further away [10]. The presence of viable bacteria indicate potential risk to exposed health workers and waste handlers in the absence of appropriate safety precautions. It has been argued that while the microbial loads in SMW may not be available in doses sufficient to cause infection, SMW generated in the community contain much higher doses and is less likely to undergo specific treatment like hospital waste [10].

In Ghana, there is a lack of uniformity in adherence to the guidelines and policy on healthcare waste. Reliance on internally generated revenue for healthcare delivery at district level and low prioritization of waste management have contributed to reluctance by health facilities to allocate funds to enable them meet standard requirements for medical waste collection and treatment. Additionally, there is limited investment by the private sector in improving existing medical waste management infrastructure. In the long term, these have contributed to different high-risk profiles for public health and the environment. Furthermore, there is limited guidance available to household members with regards to the appropriate handling and disposal of SMW generated in the absence of a health care provider who would otherwise take responsibility for it. SMW generated in households includes lancets, needles and syringes, unwanted medication and wound dressing among others. This often left to mix with household waste without consideration given to potential health risks, especially risks to unprotected waste handlers, as these waste items are believed to contain human pathogens [11]. Lack of segregation in most households, the presence of waste scavengers and informal waste porters and the proliferation of open dumpsites which are accessible to children and stray animals, potentially enhance exposure to hazards and their consequences such as community acquired needle stick injuries.

Notably in rural communities, most people are unaware that open burning of SMW contributes substantially to environmental pollution. Burning of medical waste leads to emission of air pollutants such as heavy metals, dioxins and furans which can be spread over a wide area [12]. Some of the healthcare facilities do not have functional incinerators in the respective municipalities for managing SMW generated during health care delivery. Consequently, most of these health care facilities discard medical waste at dump sites where they are subsequently burnt. This practice is environmentally unfavorable because it can potentially expose the unsuspecting public to infectious organisms before it is burnt and when it is burnt, to toxic emissions. The present study aims to detect, identify and quantify pathogens found in SMW to provide empirical evidence highlighting the need for universal implementation of appropriate medical waste disposal and treatment, irrespective of the source. While studies that quantify waste generation and composition of SMW are rife, fewer studies have focused on quantification of the pathogens present, which is potentially useful for exposure assessment in risk assessment studies. The present research was predicated upon three main objectives: (i) to identify microorganisms (bacteria) present in solid medical waste at health care facilities and households; (ii) to identify disposal methods for SMW at sub-district level health care facilities and households and (iii) to determine the factors associated with disposal of SMW among household members.

## Methods

### Research design

A cross-sectional study design was employed, with both qualitative and quantitative methods used in collection and analysis of data for the study.

### Study setting

The study was conducted in the Yilo Krobo Municipality, an administrative unit in the Eastern Region of Ghana. The municipal capital, Somanya is situated approximately 50 km from Accra, the nation’s capital. It has a population of 87,847 residing in 20,613 households based on the 2010 Population and Housing Census. Nearly seventy percent of the population live in rural communities. The municipality has 18 health care facilities including one polyclinic, six health centres and eleven community-based health planning and service (CHPS) compounds. Among these, two health centres and eight CHPS compounds were selected purposively to obtain adequate coverage of sub-district level facilities in the municipality. In addition, households within one-kilometer radius of these facilities were included in the study.

### Study population

The study population was made up of 64 health care staff from ten sub-district healthcare facilities and 385 key informants residing in households sited within one kilometre radius of the selected healthcare facilities. The health care staff comprised of clinical, auxiliary and janitorial staff. The sample size for the household survey was estimated using the Cochran formula (n = Z^2^α*pq*/L^2^) where ‘permissible error, *L* was 5%, p = 50%, *q* being *1-p*, and Zα was 1.96, being the value for standard normal variate.

Households were selected by cluster sampling from the communities where the healthcare facilities were located. One key informant was interviewed from each household. The informant was an adult aged 18 years or older, conversant with housekeeping arrangements in the household, resident in the community for at least six months and gave informed consent to participate in the study. If more than one respondent was eligible, the respondent was selected by a ballot.

For the waste stream analysis, eligible households must be sited at about 200metres to 1000metres away from a healthcare facility. This gave some assurance that SMW recovered from the household was generated by the members of the household and not litter from a nearby healthcare facility. Fifteen households were selected randomly from five communities to obtain a total of 75 samples, meeting the criterion of 1:10 suggested by [13].

### Data collection methods

Questionnaires were administered to household respondents and healthcare workers after obtaining their written consent. The questionnaire consisted of 33 items divided into four sections. Section A collected information about the respondent, Section B addressed training and management of medical waste (for healthcare care staff), Section C collected information about waste generation, segregation and storage and section D addressed disposal methods and associated outcomes. Additionally, health care workers were asked about disposal of SMW at home as an open-ended question. The purpose was to obtain their views regarding household disposal of SMW. Health facility and household samples of SMW were obtained and sent for laboratory analysis. Culture and biochemical tests were used for isolation and identification of bacteria associated with SMW respectively. Microbiological examination was performed to ascertain the type and counts of the microorganisms. The questionnaire survey was conducted during the break period at the healthcare facilities. The selected healthcare facilities had a total staff strength of sixty-four. All serving staff at the facility during the study period were interviewed. The duty rosters of the service units were used as a nominal roll.

Field visits were made to each healthcare facility to observe waste management activities at the health care facilities. Each site was visited twice every week for four weeks and observations were made using a checklist. All the sites were visited unannounced to ensure observations reflected practical reality. The observations were carried out and informal interviews held with randomly selected staff to verify information gathered through questionnaire administration.

### Sample collection

The following samples were collected and transported observing precautions enumerated in Ghana Health Service Guidelines on Health Care Waste Management, 2006: (i) samples of SMW from each health facility, (ii) a soil sample was obtained from a dumpsite where SMW was burnt, (iii) a sample of SMW recovered from household waste and (iv) residue from treated SMW taken from a functional incinerator at a polyclinic in the municipality and from an autoclave for managing medical waste operated by a medical waste management firm in Accra. In total, 15 samples were sent for the microbiological analysis.

### Sample preparation and analysis

The SMW and dumpsite soil samples were processed in conformity with the method described by [14]. One liter of sterile, distilled water was added to every 10 grams of a waste sample or item and mixed vigorously using a glass rod. The prepared sample was stored at a temperature of 25 °C (±1 °C) for 1 hour. Bacterial identification was conducted following standard procedures. A 0.1 mL aliquot of the sample was cultured on blood and MacConkey agar media to isolate, identify and count Gram positive and Gram-negative colonies, respectively. Bacterial cultures on agar media were prepared in triplicate. Single isolated bacterium colonies were obtained after sub culturing. In order to perform 16s rDNA analysis of the bacteria, the single isolated bacterium colony was transferred into nutrient broth and incubated for 18 hours with agitation at 200 rpm and appropriate aeration. The culture was pelleted through centrifugation at 1,500 × *g* for 20 min at 4 °C before the broth was discarded. QIAamp DNA Mini Kit was utilized to extract the bacterial genomic DNA according to the manufacturer’s instructions. The extracted genomic DNA was tested for integrity by quantifying its absorbance at A_260_/A_280_ using a nano-photometer. The 16s rDNA sequencing was carried out and obtained sequences were analyzed using the standard nucleotide BLAST tool (NCBI). The identities of the bacteria were determined by observing their similarity with the posted sequences. Other biochemical tests including catalase test, oxidase test, triple sugar iron test were used for the morphological analysis of bacteria. Descriptive analysis generated frequencies and percentages. Inferential analysis was performed using logistic regression analysis. Results were adjusted odds ratios and 95% confidence intervals constructed around the estimates. All analyses were performed using Stata 16.1 and a p-value≤0.05 deemed significant.

### Quality control

Two trained research assistants assisted the principal investigator with data collection. Collection of waste samples and transportation were done at temperatures that ensured the survival of microorganisms, 25 °C (±1 °C). Treated SMW and unused medical items were used for quality assurance, since microbes are ubiquitous in the environment and could potentially contaminate samples. Quantitative data collected from households and healthcare personnel were stored electronically using double entry of data and the databases compared to eliminate errors. Qualitative data and observations were captured as field notes which were summarized and shared with informants from households and healthcare staff for validation.

### Ethics statement

The study was conducted according to the guidelines of the Declaration of Helsinki, and approved by the Ethical and Protocol Review Committee of the College of Basic and Applied Sciences, University of Ghana with protocol identification number: ECBAS 067/19-20. Clearance to conduct the study was also obtained from the Municipal Health Directorate. All participants were provided information about the nature and the purpose of the study and informed written consent was obtained. Confidentiality was upheld in all interviews, healthcare facilities were de-identified and precautionary measures were observed during data collection such as the use of personal protective equipment, hand hygiene and appropriate disposal of SMW post-analysis. Recovery of SMW from the households was duly permitted by the head of the household and each household’s waste was sorted and processed in the presence of the informant. Households participating in the waste stream analysis were informed about household waste collection as part of the participant information prior to consent.

## Results

### Demographic characteristics of healthcare workers

A total of 64 respondents participated in the study from ten healthcare facilities. Respondents comprised of Nurses 48(75%), Environmental Health/Sanitary Officers 10(15.6%), Laboratory technicians 2(3.1%), and Dispensary officers 1 (1.6%) and others 3(4.7%). The mean (s.d.) age of the respondents in years was 31(±6.81). The sample was predominantly female (78.1%) and all respondents had completed secondary education (Table 1).

**Table 1:** Demographic characteristics of healthcare workers.

Characteristics	Frequency	Percentage
**Age (years)**		
20–27	21	32.8
28–32	22	34.4
33+	21	32.8
**Mean (s.d.)**	**31(±6.81)**	
**Sex**		
Male	14	21.9
Female	50	78.1
**Work Experience (years)**		
1–3	18	28.1
4–5	21	32.8
6+	25	39.1
**Mean (s.d.)**	**5(±3.91)**	
**Designation**		
Nurse	48	75
Environ. Health /Sanitary officer	10	15.6
Laboratory technician	2	3.1
Dispensary Officer	1	1.6
Others (clinical Assist.)	3	4.7

Note: s.d. = standard deviation.

### Demographic characteristics of household respondents

A total of 385 household members were involved in a questionnaire survey with 100% response rate. The mean (s.d.) age of respondents in years was 38 (±11.67) (Table 2). The sample had a predominantly female distribution 278 (72.6%), they were mostly of Ga-Dangme ethnicity 283 (73.9%), and their main occupations were farming (37.7%) and trading (30.1%). Majority of respondents had either no formal education 138 (36.0%) or had only basic education 112 (29.2%) (Table 2).

**Table 2:** Characteristics of household respondents.

Demographic	Frequency	Percent
**Age (years)**		
20–30	113	29.4
31–40	106	27.5
41–50	104	26.5
51+	62	16.6
**Mean (s.d.)**	**38(±11.67)**	
**Sex**		
Male	107	27.8
Female	278	72.2
**Education background**		
No Formal Education	138	35.8
Basic	112	29.1
Secondary	80	20.8
Tertiary	55	14.3
**Occupation**		
Civil service	14	3.6
Public service	57	14.8
Farming	145	37.7
Trading	116	30.1
Others	53	13.8
**Ethnicity**		
Ga/Dangme	284	73.8
Akan	60	15.6
Ewe	41	10.6
**Years lived at this residence mean (±s.d.)]**	[33(±15.20)]	

Note: s.d. denotes standard deviation.

### Diseases associated with solid medical waste reported by household respondents

Responses were solicited from household respondents about diseases associated SMW (Table 3). Respondents were permitted to mention up to five diseases, but most gave three responses or less. Results for each disease were presented as a relative proportion of the total responses and the relative proportion of respondents who listed it. The top five diseases listed were cholera/diarrhea 127(33%), tetanus 51(13.2%), HIV/AIDS (11.7%), respiratory tract infections (e.g. pneumonia, asthma etc.) (10.4%) and Tuberculosis (10.1%).

**Table 3:** Diseases associated with solid medical waste as reported by household respondents.

Type of disease	Number of responses	Percentage (%)
Cholera/diarrhea	381	83.0
Cough/flu/catarrh	79	21.0
Covid-19	100	26.0
TB	117	30.0
Hepatitis B	59	15.0
HIV/AIDS	136	35.0
Other LRTI (pneumonia, asthma)	74	19.0
Skin disease (measles, chickenpox)	24	6.0
Tetanus	153	40.0

### Solid medical waste recovered from households

SMW recovered from household waste comprised of sharps waste (knife, blade, infusion set, broken glass, syringes and needles), non-sharps infectious waste (cotton swabs, gauze bandages, plaster, soiled examination gloves), pharmaceutical waste (expired and used drugs) and discarded plastics containing medication.

### Bacteria isolation in solid medical waste

Bacteria were detected in waste samples from healthcare facilities and households (Table 4). Bacteria detected in SMW generated at a healthcare facility were: *Bacillus subtilis*, *Klebsiella pneumonia*, *Pseudomonas aeruginosa*, *Clostridium tetani*, *Enterococcus faecalis*, *Acinetobacter spp*. *Escherichia coli and Enterococcus faecium*. The microbial load ranged from 0.046 x 10^3^cfc/mg to 0.167 x 10^3^cfc/mg in Community-based Health Planning and Services (CHPS) compounds and 0.036 x 10^3^ to 0.071 x 10^3^cfc/mg in Health Centres, which are a higher level of healthcare service delivery. Some of the bacterial species were isolated from SMW generated in households such as *Enterococcus faecalis*, *Klebsiella pseudomonas*, *Escherichia coli*, *Bacillus cereus and Enterococcus spp*. Microbial load in SMW in households were relatively higher and ranged from 0.118 x 10^3^ to 0.125 x 10^3^ cfc/mg. Treated medical waste samples and unused medical items were also sampled for analysis and *Bacillus spp*. and *Pseudomonas spp*. were detected (Table 4). Bacteria identification was carried out on soil samples recovered from dumpsites where SMW was discarded and one soil sample collected from the surrounding (outside the healthcare facility’s environment). *Escherichia coli*, *Citrobacter spp*. *Bacillus subtilis*, *Enterococcus spp*. *and Pseudomonas stutzeri* were found in dumpsite soil, whereas *Escherichia coli* and *Bacillus spp*. were detected in the soil sample taken from outside of the healthcare facility. Microbial load ranged from 0.104 x 10^3^cfc/mg to 0.295 x 10^3^ cfc/mg in soil samples. Treated SMW had the lowest bacteria counts relative to other sources of SMW, followed by samples from health centres. The highest bacterial count (0.295 x 10^3^ cfc/mg) was obtained from a soil sample taken from a medical waste dump (Table 4). With the exception of *Klebsiella pneumoniae*, *Acinetobacter spp*. and *Clostridium tetani* found exclusively in untreated solid medical waste generated in the healthcare facilities, other species of bacteria were similar to those found in the environment and in a sample of treated solid medical waste. Some organisms, namely *Klebsiella spp*. *Acinetobacter spp*., *and Pseudomonas aeruginosa*, remained viable after several days.

**Table 4:** Bacteria detected in solid medical waste and soil from medical waste dump sites.

NO	SAMPLE	TIME (hours)	MAXIMUM COUNT (cfc/mg)	ISOLATE(S)
1	CMS_1_	5	0.167 x10^3^	Bacillus spp., Klebsiella spp., Pseudomonas stutzeri
2	CMS_2_	5	0.143 x10^3^	Pseudomonas spp., Clostridium tetani, Bacillus spp.
3	CMS_3_	5	0.046 x 10^3^	Bacillus spp., Enterococcus spp.
4	HMS_1_	5	0.044 x 10^3^	Bacillus spp.
5	HMS_2_	5	0.071 x 10^3^	Acinetobacter spp., Pseudomonas spp.
6	HMS_3_	5	0.036 x 10^3^	E. coli, Enterobacter spp., Bacillus spp.
7	HHS_1_	5	0.122 x 10^3^	Bacillus spp., Enterococcus spp.,
8	HHS_2_	4	0.125 x 10^3^	E. coli, Bacillus spp., Enterococcus
9	HHS_3_	4	0.118 x 10^3^	Klebsiella pseudomoniae, E. coli, Citrobacter koseri
10	HHS_4_	4	NSG	No significant growth
11	SSH_1_	5	0.295 x 10^3^	E. coli, Citrobacter spp., Bacillus spp.
12	SSH_2_	5	0.104 x 10^3^	Enterobacter spp., E. coli, Bacillus spp.
12	SSH_3_	5	0.135 x 10^3^	Bacillus spp., E. coli, Pseudomonas spp.
11	TMW_1_	4	NSG	No significant growth
12	TMW_2_	4	0.041 x 10^3^	Bacillus spp., Pseudomonas spp.
13	TMW_3_	4	NSG	No significant growth

*Key*: NSG: No significant growth, Time: delivery time, CMS = solid medical waste from CHPS compound, HMS = solid medical waste from health centres, HHS = solid medical waste generated in homes, SSH = soil obtained from medical waste dumpsites, TMW = treated medical waste item.

### Waste disposal method adopted by healthcare facility and households

The disposal methods reported in Health Centres and CHPS compounds indicate that open burning (97.5% vs. 96.3%) and burying (95% vs. 100%) were the most common methods used (Table 5). Fifteen per cent of respondents at the Health Centres reported that the Municipal Health Directorate come to collect infectious waste (sharps and non-sharps infectious waste) for incineration.

**Table 5:** Disposal practices of solid medical waste in sub district level.

Waste Disposal	Healthcare Centre (N = 40)	CHPS (N = 24)	Households (N = 385)
Burning in the open	39(97.5)	23(96.3)	113(29.4)
Burying	38(95.0)	24(100)	96(24.9)
Dumpsite	-	-	190(49.3)
Incineration	6(15. 0)	-	-
Treatment with disinfectants	1(2.5)	1(4.1)	-

Values provided represent frequency with percentage in brackets.

SMW generated in households was treated and disposed like municipal waste, therefore final disposal at the dumpsite (49.3%) was most mentioned option, followed by burning in the open (29.4%) and burying (24.9%).

### Relationship between disposal practices and socio-demographic characteristics of household respondents

In the relationship between disposal practices of households (dump site, burning and burying) and sociodemographic factors, age of the respondent independently influenced practice (Table 6). Household members aged 51 years and older were three times more likely to place SMW in household waste that was later discarded at dumpsites compared with their counterparts aged 20–30 years (aOR = 3.37; 95%CI = 1.41–8.02). This age group was less likely to burn SMW (aOR = 0.35; 95%CI = 0.12–0.99). Participants aged 41–50 years were 58% less likely to bury SMW compared with those aged 20–30 years (aOR = 0.42; 95%CI = 0.18–0.98). For burning and burying, the effects were marginal (Table 6).

**Table 6:** Relationship between disposal practices and sociodemographic characteristic of household respondents.

	Household Solid Medical Waste Disposal Practices		
	Dump Site	Burning	Burying
Demographic	aOR[95%CI], p-value	aOR[95%CI], p-value	aOR[95%CI], p-value
**Age**			
20–30	Ref	Ref	Ref
31–40	1.66[0.93–2.87]0.085	0.56[0.29–1.04]0.068	0.92[0.47–1.81]0.810
41–50	1.68[0.87–3.26]0.122	1.30[0.66–2.55]0.444	0.42[0.18–0.98]0.045*
51+	3.37[1.41–8.02]0.006*	0.35[0.12–0.99]0.047*	0.49[0.17–1.45]0.200
**Sex**			
Male	Ref	Ref	Ref
Female	1.18[0.72–1.93]0.518	1.04[0.60–1.78]0.896	0.73[0.42–1.29]0.282
**Education background**			
No Formal Education	Ref	Ref	Ref
Basic	1.02[0.59–1.77]0.937	0.86[0.46–1.63]0.656	1.06[0.57–1.97]0.854
Secondary	1.07[0.55–2.08]0.844	0.98[0.47–2.02]0.952	0.82[0.36–1.83]0.621
Tertiary	1.00[0.34–2.96]0.999	1.10[0.36–3.36]0.871	1.11[0.31–3.98]0.877
**Occupation**			
Civil service	Ref	Ref	Ref
Public service	0.75[0.20–2.77]0.665	1.27[0.32–4.99]0.727	1.20[0.22–6.55]0.834
Farming	0.56[0.14–2.23]0.409	0.79[0.18–3.39]0.754	2.53[0.43–14.77]0.302
Trading	1.07[0.27–4.21]0.926	1.01[0.24–4.23]0.987	1.20[0.21–7.07]0.837
Others (Mason, mechanics, technicians etc.)	1.16[0.28–4.88]0.836	1.14[0.25–5.06]0.867	1.11[0.17–7.13]0.909
**Ethnicity**			
Ga/Dangme	Ref	Ref	Ref
Akan	1.32[0.73–2.38]0.359	0.76[0.39–1.46]0.406	0.84[0.40–1.76]0.639
Ewe/Guan	0.59[0.28–1.22]0.152	0.95[0.44–2.07]0.896	1.81[0.82–3.95]0.139
**Years lived at this residence**	0.99[0.98–1.02]0.901	0.99[0.97–1.01]0.409	1.02[0.99–1.05]0.183

*Statistically significant at p≤0.05; marginal effects of age for burning and burying.

## Discussion

### Bacteria detected in solid medical waste at health facility

Solid medical waste (SMW) generated in healthcare facilities is believed to contain pathogenic microorganisms [15, 16]. To ensure safety, knowledge about microbial content is essential, including the survival characteristics of microorganisms inside SMW. The present study demonstrated that pathogenic bacteria were isolated from used syringes/needles, soiled gauze bandages, surgical gloves, plasters and glass ware generated at the healthcare facility. The result demonstrated the presence of bacteria namely *Pseudomonas aeruginosa*, *Cinetobacter spp*., *Klebsiella pneumonia*, *Clostridium tetani*, *Enterococcus faecalis*, *Escherichia coli*, *Bacillus subtilis*, *Bacillus cereus and Citrobacter koseri*. These bacteria have also been isolated from hospital SMW and reported by other authors [16–19]. *Enterococcus faecalis* and *Enterococcus faecium* were the most common species isolated. *Enterococcal species* have been isolated from untreated and treated hospital waste from three European countries as reported by [20], and this is consistent with the present study. Similarly, [21] in another study, investigated microbial loads and compared microbial survival in general hospital waste with infectious waste. Bacteria isolated from SMW in their study were *Pseudomonas spp*., *Serratia spp*., *Escherichia spp*., *Enterococcus spp*. *Streptococcus spp*. *and Acinetobacter spp*. The present study reports all these species, excluding *Streptococcus spp*. and *Serratia spp*. [21] reported that bacteria identified were initially low in quantity, but grew rapidly so that significant numbers were detected within 24 hours. In the present study, *Acinetobacter spp*. remained viable after seven days. These findings align with the recommendation that early removal of SMW within 24 hours can potentially limit environmental contamination from SMW and by extension, reduce the risk of human exposure.

The study also identified ubiquitous groups of bacteria that are of relevance to human health given their role as major causative agents of healthcare associated infections. Some organisms have the ability to survive longer in the environment, on dry surfaces and they are resistant to desiccation in spore forms, an example being *Clostridium tetani*. Spores are capable of surviving high temperature ranges and some antiseptic solutions [22]. The ability of the organisms to survive under harsh conditions, makes them well adapted to the health care environment [23]. Some species, like *Klebsiella pneumonia* and *Escherichia coli*, have the ability to demonstrate resistance to antibiotics [24, 25]. Drug resistance can lead to prolonged hospital stay and increased cost due to the need for alternative, often more expensive options of treatment. *Enterococcus spp*. and *Pseudomonas spp*. are typically harmless in healthy individuals but become opportunistic pathogens in the immune compromised mainly by causing septicaemia, pneumonia, urinary tract infections, and surgical wound infections. In healthcare settings, they are spread through improper hygiene, such as from unclean hands of healthcare workers or via contaminated medical equipment that was not sterilized. Highly resistant bacteria such as multidrug-resistant Gram-negative bacteria are the cause of high incidence rates of nosocomial infections worldwide [24].

There are also beneficial roles played by some of the organisms in the environment. For instance, *C*. *freundii* can convert nitrate or the ammonium ion to nitrite. Apart from the ecological role in the nitrogen cycle, it can also accumulate uranium (which is the basic material for nuclear technology) by building phosphate. *Citrobacter spp*., for instance, is considered particularly suitable for monitoring antibiotic resistance in the environment [26].

### Bacteria isolated from SMW generated from household

The results from the study showed that both nosocomial and opportunistic bacteria were isolated from soiled cotton, soiled gauze bandages generated during wound dressings, plasters, napkins and diapers which were recovered from household bin. These waste samples were contaminated by body fluids from patients who had wound dressings in their homes and generated SMW. Microorganisms such as *Bacillus subtilis*, *Enterococcus faecalis*, *Escherichia coli*, *Klebsiella pneumoniae*, *Bacillus anthracis* and *Citrobacter spp*. were isolated on the samples recovered. Other studies have also documented the presence of bacteria in general waste, although these were not drawn from households [16, 21].

### Isolation of bacteria from SMW dumpsite soil

Bacteria identification was carried out on soil samples recovered from a dumpsite where SMW is discarded. The report indicated that *Escherichia coli*, *Bacillus spp*., *Citrobacter freundii*, *Enterococcus spp*. and *Pseudomonas stutzeri* were isolated in the soil. This is consistent with a study by [27] that reported isolation of *Enterobacter spp*., *Bacillus spp*., *Escherichia coli*, *Klebsiella spp*., *enterococcus spp*., *Pseudomonas spp*., *Serratia spp*. from a dumpsite. *Bacillus spp*. were reported to have been detected in soil at dumpsites in a study by [15]. A study by [28] reported that mutant *E coli* is capable of growing at 65°C. The ability to survive high temperatures may suggest that pathogens in SMW residue at dumpsites still have the potential to transmit diseases [29]. Pathogenic *Bacillus spp*. isolated at the dumpsite are capable of causing life-threatening infectious diseases such as pneumonia, meningitis and toxic shock syndrome. *Enterococcus spp*. is a nosocomial opportunistic pathogen which has been associated with bacteremia, urinary tract infections and meningitis in humans. Citrobacter spp. was one of the bacteria isolated from the SMW sample with the highest microbial load. Citrobacter spp. have been associated with antibiotic resistance, particularly Ampicillin [30]. Infections arising from inadvertent exposure may be more difficult and expensive to treat.

### Disposal methods of SMW in households

The most commonly used method for disposal of SMW by members of households in rural communities as reported in this study was discarding at a dumpsite. Disposal of used cotton, used gauze, expired or unused medicines, soiled bandages and plaster in the household bin mingled with household waste and later discarded at the dumpsite, has also been reported in some parts of the world [31, 32]. Syringes and needles, medication bottles and saline bags were found at dumpsites located near homes and it was feared that children could get injured and possibly be infected with pathogens as they play with discarded medical items. Similar fears were expressed in Botswana as reported by [33], where caregivers expressed concern about children playing with syringes and needles they found at dumpsites. Apart from the injury that sharps can cause to these children, they could pick up pathogens as they touch waste items in the environment.

SMW mixed with general waste in healthcare facility, is assumed to have its waste load contaminated if there is no segregation [1, 34]. The co-mingling of medical waste with other types of waste in homes could lead to cross contamination, where pathogens from SMW are transferred to household waste and vice versa. According to standard practice, the SMW should be treated to reduce the volume as well as render the waste relatively less harmful before it is discharged at a landfill or dumpsite. In spite of the pathogenic nature of SMW generated in homes, less attention is given to it compared to that generated in healthcare facilities. Household bins which contain waste including SMW are often kept in the open without a cover, which allows easy dispersal by scavenging animals.

Burning has been reported as one of the most preferred disposal options in households for managing SMW. Burning of SMW as reported was mostly done at the backyard. Complete burning of SMW is not assured through this practice. Incomplete combustion during backyard burning contributes significantly to pollution of the environment [35–37]. Smoke containing heavy metals emitted from SMW during open burning are discharged in the environment, exposing unsuspecting persons to contaminants which could contribute to respiratory diseases. In UK and Sweden, people are encouraged to return their expired or unused medicines through pharmacies to be incinerated at high temperatures [38]. A study conducted on a retrieval system for expired or unused medicines in Ghana suggested less than 5% of the respondents were willing to return their unwanted medicines to be incinerated [39]. This aligns with the finding in the present study that it was more convenient for households to either bury or burn.

### Disposal methods in healthcare facility

The most common disposal method reported in this study is burying which is usually done in a dugout pit. Dumping in pit (burying) is one of the most preferred methods of disposing SMW in Africa [40]. This was reportedly a less expensive method and convenient. However, the method has been known to be a potential source of soil and water contamination. Unrestrained disposal of SMW such as dumping in shallow pits make waste accessible to waste pickers and animals [34, 41]. Leachate from buried SMW introduces contaminants into ground water [41, 42].

Incineration has been recommended as a short-term method for treating SMW, especially infectious waste and sharps wastes. The high-temperature application during incineration process ensures complete combustion and changes the waste into ashes which can be discarded at a designated landfill. At sub-district level, SMW is burnt at dumpsites, because they do not have incinerators. This act of burning SMW openly potentially contributes to air pollution in these areas. The sub-standard methods of SMW disposal has been described in other developing countries. For instance, [4] reported that only 45% of hospitals had adequate disposal systems for SMW.

### Factors that affect disposal practices at household level

SMW disposal at dumpsites by household members was associated with older age. People aged 51 years and older preferred to discard SMW at a dumpsite (aOR [95%CI], 3.37[1.41–8.02]) compared to those aged 20–30 years. Older persons are more likely to have chronic conditions resulting in generation of SMW. Since households often lack clear options for disposal of SMW, an older adult or caregiver could find it convenient to place SMW in household waste that is eventually discarded at dumpsites. On the other hand, younger persons are more likely to have medical procedures performed in the hospital setting, where SMW generated can be adequately managed.

### Future directions of research and recommendations

Future studies are required to determine the survival of pathogens from SMW under prevalent disposal methods and environmental conditions to ascertain acceptable on-site methods of handling and disposal from households. In the interim, available local evidence can be used to develop public guidelines in management of SMW generated in households. Current guidance places responsibility for SMW on healthcare and alternative practitioners, but family members may also be involved in generating SMW and require adequate guidance. Households should demand this guidance from relevant agencies through public fora. Stakeholders in healthcare waste management can dialogue with members of the public to enlighten the public about appropriate ways to collect and manage SMW generated at home using broadcast media for a wide reach. Monitoring to ensure compliance with existing SMW management protocols should be revisited at sub-district level healthcare facilities.

### Study limitations

The study involved ten sub-district level healthcare facilities and the findings might differ from higher level healthcare facilities because of the scale of services offered and patient attendance. Additionally, the authors acknowledge that the presence of pathogens in SMW does not necessarily translate to harm to human health. The consistent use of personal protective equipment, appropriate treatment and hand hygiene can help minimize exposure. However, previous studies suggest that the unsuspecting public, unsupervised children and inadequately protected scavengers, may be exposed, enhancing the potential for harm [43–45]. Based on this, we place our findings and recommendations within the domain of the precautionary principle which states that if a product, action or a policy has a suspected risk of causing harm to the public or to the environment, protective action should be supported before there is complete scientific proof of a risk.

## Conclusion

SMW was generated in households and respondents reported categories similar to those in sub-district level healthcare facilities in a multi-phased study in Yilo Krobo Municipality, Ghana. The study confirmed the presence of pathogenic bacteria in SMW. Bacteria isolated in healthcare facilities were: *Bacillus subtilis*, *Klebsiella pneumonia*, *Pseudomonas aeruginosa*, *Clostridium tetani*, *Enterococcus faecalis*, *Acinetobacter spp*., and *Escherichia coli*. *Klebsiella pneumoniae*, *Citrobacter spp*., *Escherichia coli*, *Bacillus cereus and Enterococcus spp*. were isolated in SMW generated in households. *Pseudomonas stutzeri*, *Escherichia coli*, *Citrobacter freundii*, *Bacillus spp*. *and Enterococcus spp*. were detected in soil recovered from a dumpsite where SMW was discarded. Empirical evidence has been provided to justify precautionary measures when handling SMW. SMW disposal practices were sub-optimal in sub-district healthcare facilities. This highlights the need for institutionalizing appropriate treatment methods in sub-district level facilities and/or strengthening the linkages with higher level facilities to ensure regular removal of SMW. In households, the most prevalent methods of disposal were dumping in pits and burning, which can cause injury hazards and air pollution respectively. Strict adherence to appropriate waste management practices at sub district level health facilities should be enhanced by monitoring and training. Further research is needed to identify environmentally safe ways for households to manage SMW left in their care and should culminate in a public guidance document. These measures will insulate the public from potentially hazardous environmental conditions that pose a threat to human health.
